# Leptin and PCSK9 concentrations are associated with vascular endothelial cytokines in patients with stable coronary heart disease

**DOI:** 10.1515/med-2021-0400

**Published:** 2022-01-18

**Authors:** Qiang Wang, Bo Zheng, Peng Chen, Yan Lei

**Affiliations:** Department of Cardiovascular Medicine, Wangjing Hospital, China Academy of Chinese Medical Sciences, Chaoyang District, Beijing 100102, China; Department of Cardiovascular Medicine, Affiliated Hospital of Binzhou Medical University, BinZhou City, China; Department of Molecular Biology, Beijing Key Laboratory of Traditional Chinese Medicine Basic Research on Prevention and Treatment for Major Diseases, Experimental Research Center, China Academy of Chinese Medical Sciences, Dongcheng District, Beijing 100700, China

**Keywords:** coronary heart disease, vascular inflammation, leptin, PCSK9

## Abstract

Leptin and proprotein convertase subtilisin kexin 9 (PCSK9) play an important role in regulating blood lipid concentration. Recently, they have been found to show the ability to independently regulate the immune response. Vascular immune response has an important pathological function in the development of coronary heart disease (CHD) and thrombosis. The aim of this study was to explore the relationship between leptin, PCSK9, and vascular endothelial cell related inflammatory factors. First, detailed clinical information were collected and analyzed for 27 patients with stable CHD and corresponding 27 healthy controls. Second, using liquid-phase protein chip technology, leptin, PCSK9, and vascular-related inflammatory factors, such as E-selectin, vascular cell adhesion protein 1 (VCAM-1), intercellular cell adhesion molecule-1 (ICAM-1), interferon-gamma (IFN-γ), and interleukin-17 (IL-17), were detected on the same platform. Finally, the correlation between leptin, PCSK9, and the inflammatory factors was analyzed. Through collecting clinical information of patients, it was suggested that there was a significant positive correlation between leptin and blood lipid level in CHD. Compared with healthy people, the levels of leptin, PCSK9, E-selectin, and ICAM-1 were significantly high in patients with CHD. There was a high positive correlation between leptin and E-selectin, ICAM-1, IFN-γ, and IL-17. Also, a high positive correlation between PCSK9 and E-selectin, IFN-γ, and IL-17 concentrations was observed. In general, leptin and PCSK9 may not only be able to regulate lipid metabolism, but may also be able to regulate inflammation in CHD.

## 1 Introduction

Leptin is a protein secreted by adipose tissue, which is widely believed to participate in the regulation of glycometabolism, fatty acid, and energy metabolism. It can reduce food intake, increase energy release, inhibit the synthesis of adipocytes, and then reduce weight. The recent studies found that leptin can also regulate immune inflammatory response [1,2,3,4]. In the innate immunity, leptin promotes natural killer cell activation, neutrophil chemotaxis, and macrophage secretion of tumor necrosis factor-alpha (TNF-α), Interleukin-6 (IL-6), and Interleukin-12 (IL-12). In the adaptive immunity, leptin stimulates the proliferation of immature T cells and inhibits CD4^+^CD25^+^Foxp3^+^ regulatory T cells. Importantly, researchers found that leptin increased the differentiation of Th17 cells in Systemic Lupus Erythematosus patients [5,6]. However, it is still unclear whether leptin is associated with vasculitis endothelial cell inflammation in patients with coronary heart disease (CHD).

Proprotein convertase subtilisin kexin 9 (PCSK9), as a neuronal apoptotic regulatory invertase, not only participates in liver regeneration and regulates neuronal apoptosis, but also affects low-density lipoprotein (LDL) internalization by reducing the number of LDL-receptor (LDLR) on hepatocytes, so that the LDL in blood cannot be cleared, leading to hypercholesterolemia [7]. PCSK9 is a central participant in atherosclerosis and a key immune effect of oxidized LDL inducing dendritic cell maturation and plaque T cell activation [8]. This may directly affect atherosclerosis and cardiovascular disease.

Vascular endothelial cell inflammation is the key node of thrombosis, and adhesion molecules play a decisive role in the early stage of thrombosis [9]. There are few reports on the correlation of leptin, PCSK9, and CHD vascular inflammation in CHD patients. So, the aim of this study is to explore the relationship between serum leptin, PCSK9, endothelial cell adhesion factors, and Interleukin-17 (IL-17) levels in CHD patients. This study will help to clarify whether PCSK9 and leptin are involved in vascular inflammation, and not just play a pathological role in CHD by regulating blood lipids.

## 2 Material and methods

### 2.1 Samples and baseline

The study was ethically reviewed by the Medical Experimental Center of the Chinese Academy of Traditional Chinese Medicine, and informed consent was issued by each patient. Samples and Healthy controls were obtained from Wangjing Hospital, Chinese Academy of Traditional Chinese Medicine and Affiliated Hospital of Binzhou Medical University. Serum samples and clinical information of 27 patients with CHD and 27 healthy controls were collected in this study. Diagnosis of CHD was accomplished by coronary Computed Tomography or coronary angiography. There was no significant difference in age and sex between the two groups.

### 2.2 Liquid-phase protein chip

Liquid-phase protein chip technology was used to detect the level of protein markers in this study. The specific methods were described as follows. Commercial test kits were purchased from RD Biotechnology Company, which can be used to detect seven factors including Leptin, PCSK9, Interferon-gamma (IFN-γ), IL-17, E-selectin, vascular cell adhesion protein 1 (VCAM-1), and intercellular cell adhesion molecule-1 (ICAM-1). First, the serum was mixed with the magnetic beads coated with antibodies. The diluted volume of the serum was 1:2, and the reaction was carried out at room temperature for 1 h and then washed 3 times with a magnet washer for 5 min each time. The second antibody corresponding to the 7 proteins was added and reacted at room temperature for 1.5 h. Fluorescent pigments were added and reacted at room temperature for 10 min. After cleaning, the fluorescence value was read by an instrument, and the concentration of the sample was calculated according to the standard curve.

### 2.3 Statistics

Differences in clinical information and protein concentration among different ethnic groups were examined by *t*-test. All the drawings in this article were executed by Microsoft Excel. The fitting of the standard curve for protein concentration determination was based on 4PL method. *P* value less than 0.05 was considered to have significant statistical difference.

## 3 Results

### 3.1 Clinical parameters and concentration of leptin and PCSK9

First, 27 stable CHD patients (62.52 ± 10.38 years old; 15 males and 12 females) and 27 healthy controls (60.0 ± 11.24 years; 15 males and 12 females) were recruited (Table 1). There was no statistical difference (*P* < 0.05) in age and gender data between the two groups. The male patients with CHD were more than the female patients. Male patients with stable CHD were younger than female patients (*P* < 0.05). The LDL-C, CHO, apolipoprotein B (Apob), and apolipoprotein A1 (Apoa1) levels of female patients with stable CHD were higher than that of male patients, and they were statistically significant (*P* < 0.05). There was no significant difference in other indexes between the two groups (*P* > 0.05). It showed that female had higher risk of CHD than male. According to the standard curve of 65 years old, CHD patients were divided into two groups. There was no difference in the factors between the two groups (*P* > 0.05).

**Table 1:** Clinical information of patients with CHD

No.	Gender	Age	LDL-C	CK-MB	CHO	Apob	Apoa1	HDL-C	TG	CRP	Sd-LDL	THCY
1	M	68	2.39	12.5	4.19	0.92	1.25	1.36	1.85	71.41	205	18.42
2	F	79	1.37	10.9	2.62	0.51	1.16	1.1	0.89	1.45	145	14.93
3	F	64	3.59	10.8	5.26	1.26	1.44	1.39	1.93	19.5	360	9.95
4	M	66	1.77	16.8	3.12	0.76	1.12	1.06	1.39	1.38	211	17.82
5	M	55	3.94	12.8	5.15	1.37	1.14	1.02	1.72	0.27	509	13.55
6	F	66	1.48	14	3.02	0.71	1.2	0.84	2.55	0.63	287	10.64
7	M	57	2.04	23.9	3.04	0.74	0.87	1.04	0.47	7.06	126	27.26
8	F	76	1.62	8.7	3.17	0.66	1.18	1.12	1.66	0.82	149	17.26
9	F	75	3.04	16.8	5.28	1.19	1.63	1.86	1.74	21.46	301	19.3
10	M	72	2.01	16.5	3.07	0.79	1.12	1.02	1.04	0.26	256	14.37
11	M	34	2.01	12	3.35	0.79	1.27	1.04	1.95	0.92	285	12.6
12	M	58	1.37	15.2	2.43	0.61	0.92	0.92	0.73	1.3	165	12.26
13	M	45	1.63	19.2	3.05	0.58	1.45	1.38	0.79	1.23	141	10.86
14	M	55	1.73	12.8	3.16	0.63	1.33	1.41	1.12	0.23	184	12.66
15	F	65	4	10.8	5.22	1.32	1.22	1.17	1	2.51	228	10.83
16	F	59	2.61	12.2	3.62	0.96	0.83	0.67	2.1	2.22	425	7.31
17	M	65	2.03	1.1	3.55	0.85	0.96	1.08	1.59	2.18	260	1.25
18	F	63	3.36	25.1	4.5	1.05	1.34	1.23	1.14	1.35	304	22.81
19	M	64	2.31	20.8	3.59	0.86	1.21	1.27	0.88	20.25	218	12.29
20	F	55	1.9	13.2	3.51	0.79	1.49	1.26	2.15	2.38	300	9.53
21	M	58	1.26	9.9	2.5	0.51	1.1	0.94	2.07	0.41	189	14.11
22	F	65	2.78	13.2	5.1	1.21	1.6	1.24	4.24	2	623	12.53
23	M	44	1.97	10.9	2.93	0.74	0.84	0.85	1.09	0.61	186	16.88
24	F	76	2.11	24.4	3.56	0.76	1.5	1.55	1.08	0.28	199	14.4
25	F	64	3.93	15.9	5.23	1.33	1.25	1.25	1.08	0.48	317	12.26
26	M	73	1.45	7.3	2.58	0.62	1.02	0.84	2.06	0.81	209	10.54
27	M	67	1.7	28.2	2.8	0.64	0.64	1.04	1.03	0.23	187	12.29
Unit	—	—	mM	U/L	mM	g/L	g/L	mM	mM	mM	mg/L	µM
Mean		62.52	2.27	14.66	3.65	0.86	1.19	1.15	1.53	6.06	258.11	13.66

LDL-C: Low-density lipoprotein cholesterol; CK-MB: Creatine kinase myocardial Isoenzymes; CHO: Total cholesterol; Apo: Apolipoprotein; Glu: Glucose; HDL-C: High-density lipoprotein cholesterol; TG: Total triglycerides; CRP: C-reactive protein; Sd-LDL: Small and dense low density lipoprotein; THCY: Homocysteine.

### 3.2 Association studies of endothelial cell adhesion factors with leptin and PCSK9 in CHD patients

**Figure 1:**
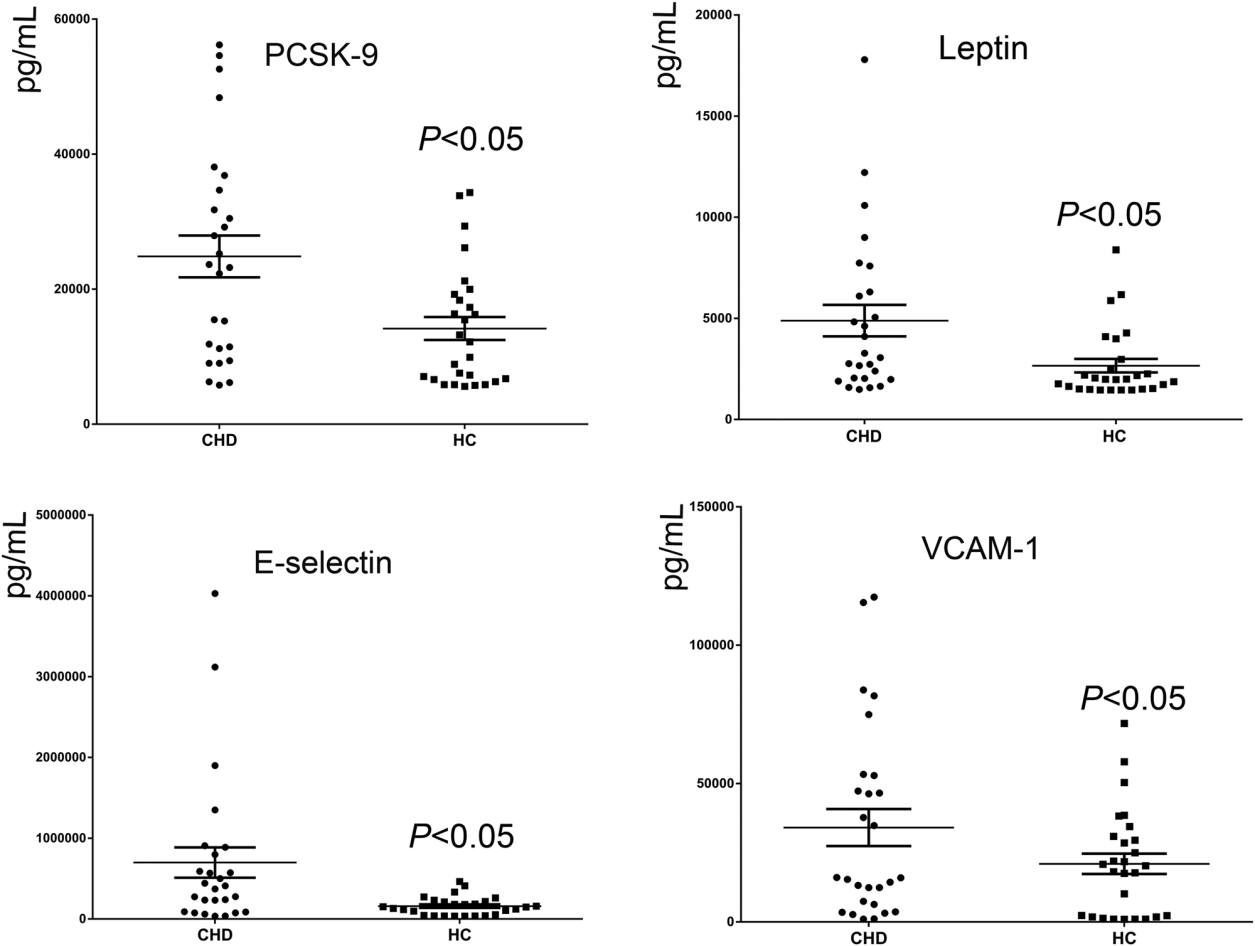
Endothelial cell adhesion factors in patients with CHD was significantly higher than that in healthy controls: PCSK9 level in CHD and HC subjects; leptin; E-selectin; and VCAM-1. CHD: coronary heart disease; HC: Healthy controls.

**Figure 2:**
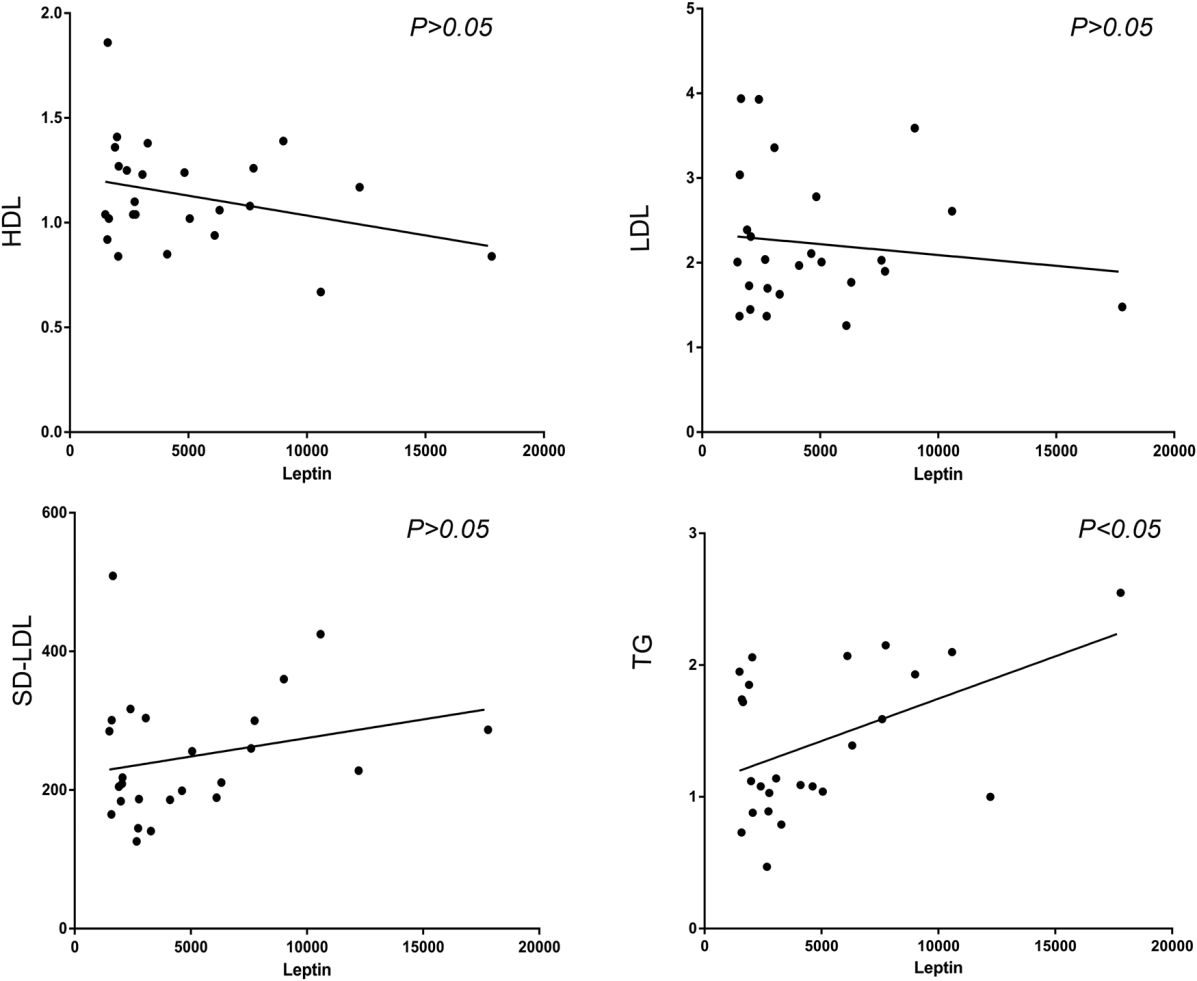
The levels of leptin were positively correlated with blood TG levels. TG: total triglycerides.

**Figure 3:**
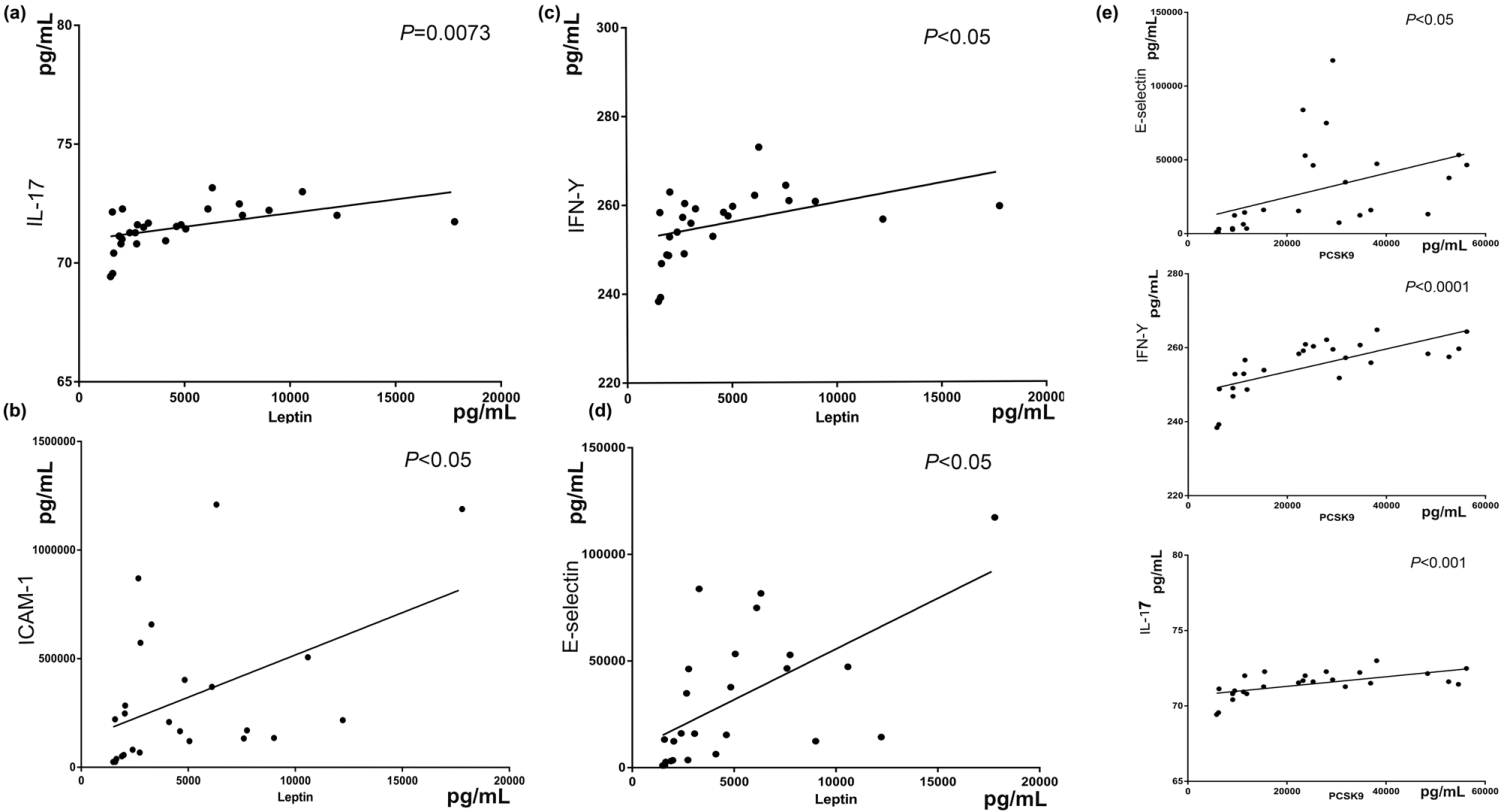
Significant relationship between leptin, PCSK9, and endothelial cell adhesion factors was observed. Correlation lines between leptin and IL-17 (a), IFN-γ (b), ICAM-1 (c), E-selectin (d); and Correlation lines between IL-17, E-selectin, IFN-γ, and PCSK9 (e).

Endothelial cell adhesion inflammation factors in patients with CHD was significantly higher than healthy controls (*P* < 0.05). The results are shown in Figure 1. PCSK9 and leptin were also highly expressed in CHD patients, and they may be related to lipid regulation and metabolism, there was also a significant relationship between leptin and blood lipid level. The levels of leptin were positively correlated with blood total triglycerides (TG) levels (Figure 2). There was a significant relationship between PCSK9 and E-selectin, IFN-γ, and IL-17. Also, there was a significant relationship between leptin and endothelial cell inflammatory factors (ICAM-1, E-selectin, and IFN-γ) and IL-17 (Figure 3). However, the specific mechanism between them and the inflammatory factors, especially endothelial cells inflammation, is still unclear.

## 4 Discussion

Leptin is a fat cytokine secreted mainly by white adipose tissue and involved in a variety of pathophysiological processes, including maintaining energy balance, regulating immune and inflammatory responses, and bone metabolism [10]. Leptin is an important intermediary for the interaction between nutritional status and neuroendocrine-immune function *in vivo* [11], and the content of Leptin is closely related to the occurrence and development of various autoimmune diseases. Leptin is an inflammatory cytokine which was involved in the pathogenesis of rheumatoid arthritis and may be an indicator of disease activity and joint failure, as well as a potential therapeutic target [12,13]. Therefore, the role of leptin as a new type of immunomodulatory agent in inflammatory response has attracted more and more attention. In this study, It was suggested that leptin also participates in the inflammatory process of vascular CHD.

PCSK9, as a neuronal apoptotic regulatory invertase, participates in liver regeneration and regulates neuronal apoptosis, and affects LDL internalization by reducing the number of LDLR on hepatocytes. As a serine protease, PCSK9 not only can degrade LDLR and elevate LDL cholesterol level in blood, but also has many other biological functions, such as participating in nervous system development, neuronal apoptosis, regulating sodium channel, islet cell function, etc. [7]. PCSK9 is produced by synthesizing PCSK9 zymogen in endoplasmic reticulum, which reacts with self-catalysis in endoplasmic reticulum or golgi body, releasing properties to form mature protease, and secreting into blood immediately. Elevated LDL cholesterol is a major risk factor for atherosclerotic cardiovascular disease. PCSK9, a new generation drug target of lipid-lowering frontier drug, provides a new therapeutic model to combat LDL cholesterol [14]. It is a great progress for lowering lipid after statins, especially in patients with statin intolerance, resistance, and familial hereditary hypercholesterolemia. In fact, PCSK9 inhibitors not only reduce LDL cholesterol but also reduce cardiovascular risk in terms of anti-inflammation according to a Phase III clinical study conducted by Amgen in March 2018 [15]. There was a significant relationship between PCSK9 and E-selectin, IFN-γ, and IL-17. This suggests that leptin and PCSK9 may independently participate in the development of CHD through inflammation regulation. They have potential value as independent indexes for evaluating inflammation and risk assessment of stable CHD.

Inflammation and inflammatory factors have been continuously concerned in the pathogenesis of cardiovascular disease; the plasma levels IL-9 was detected to be associated with cardiopulmonary dysfunction and all-cause mortality in chronic heart failure [16,17]. CHD is a pathological process characterized by chronic inflammation. The occurrence and development of CHD is always accompanied by inflammation [18,19]. Inflammatory mediators such as ICAM-1, VCAM-1, and E-selectin have been proved to play an important role in the upstream of CHD process, significantly increasing the risk of plaque progression, instability, and cardiovascular events [20]. This study explored the relationship between leptin, PCSK9, endothelial cell-related inflammatory adhesion factors, and IL-17. The results suggested that there was a correlation between them. This provided direct evidence for their association with vascular endothelial cells and autoimmunity, and provided a possible theory for guiding the use of their inhibitors. As an autoimmune-related inflammatory factor, IL-17 levels in serum of patients with CHD were not significantly different from those of healthy people, suggesting that the autoimmune properties of CHD were not significant. However, there are still some limitations in this study, such as the small number of samples and the lack of multi-center dynamic research, which is the next research we need to do.
